# Elderly and technology tools: a fuzzyset qualitative comparative analysis

**DOI:** 10.1007/s11135-016-0390-6

**Published:** 2016-07-23

**Authors:** Rana Mostaghel, Pejvak Oghazi

**Affiliations:** 1 Department of Marketing, Linnaeus University, 351 95 Vaxjo, Sweden; 2 Department of Accounting and Logistics, Linnaeus University, 351 95 Vaxjo, Sweden

**Keywords:** Senior technology acceptance model, Gerontechnology, Health and ability characteristics, QCA

## Abstract

The number of senior citizens is growing globally and governments are striving to find innovative solutions to deal with complex care demands of this part of the population. Technology has been an answer to this situation; however, it is very important that the elderly accept and actually use the technology. This paper empirically tests the senior technology acceptance model using the fsQCA method to analyse data with a sample of 811 seniors aged 60 and over living in Sweden. The results revealed that the necessary conditions for high “perceived ease of use” and “perceived usefulness” are gerontechnology self-efficacy, gerontechnology anxiety, and cognitive abilities; however, each of these is not sufficient on its own. Self-reported health conditions and physical function also play a peripheral role in achieving the desired outcome. Theoretical and managerial implications are discussed at the end of the paper.

## Introduction

The elderly population has increased tremendously over the past decades and people are living longer all around the globe. This trend has several consequences, such as increase of costs for complex care demands in different societies, for instance, in the USA (Rahtz and Sirgy 2000), the Netherlands (Peetom et al. 2015), and China (Chen and Chan 2014). Thus, solutions are needed to satisfy elderlies’ needs in their own homes as much as possible. Aging leads to an increased risk for development of chronic disorders, such as Alzheimer’s disease. In addition, the risk of falling and sustaining a hip fracture show growth among this group. All these general reductions in health condition, plus immobility, jeopardize elderlies’ independent lifestyle at their own homes (Peetom et al. 2015).

Many branches of the government look for solutions to improve the lifestyle of the elderly in their own homes, as sources for elderly healthcare are becoming scarce. Technology has been recognized as a solution to increase and support the independency and wellbeing of elderlies at home. A more efficient healthcare system is achievable through advances in tele-health, distance monitoring, and other technology tools that allow a longer independent life at home.

In 1995, Celler et al. (1995) introduced tele-monitoring systems; however, in 2016 we still do not see the everyday use of these systems. Qualitative studies have examined the elderly’s attitudes toward assistive technology (Skymne et al. 2012). Findings revealed that the elderly do not believe that technology can significantly improve their life quality; therefore, they are not ready to use these solutions (Heart and Kalderon 2013). Investing thousands of euros in technology without using it would be waste of time and resources; thus, it is highly vital to understand technology acceptance among the elderly.

This study investigates the impact of gerontechnology characteristics (i.e., cognitive abilities, gerontechnology self-efficacy, gerontechnology anxiety, self-reported health condition, and physical functioning) on perceived usefulness and perceived ease of use. Gerontechnology refers to technology tools and services that assist elderlies to offer a more independent, healthy, comfortable, safe, and socially engaged life (Lesnoff-Caravaglia 2007). In order to analyse the empirical data, the fsQCA method is employed.

The context of this study is Sweden, for several reasons. First, Sweden is second in Europe in the proportion of their population that is aged 80 years or more, approximately 5 % of the population (Sweden.se). Second, in 2010 the cost of elderly care was SEK 95.5 billion, and the private section financed only 3 % of these costs (Sweden.se). Forecasts show that in 20 years’ time, one Swede in four will be over 65 years old (homeinstead.com). Third, the European Innovation Scoreboards project for the European Commission 2015 reveals that Sweden has been a leader in innovation and technology in Europe since 2007. Finally, a systematic literature review in Swedish context emphasizes the need for a quantitative study based on a solid theoretical model on elderly acceptance of technology (Mostaghel 2016).

The rest of the study proceeds as follows. Section 2 illustrates an extensive literature review about various aspects of age-related changes and barriers for elderly using technology. In addition, this section presents the conceptual framework along with its components. Section 3 discusses the methodology, including size and nature of the sample, plus proposition testing and results. Finally, Sect. 4 explains conclusions and implications.

## Literature review

As humans age, the body undergoes age-related changes, such as visual, auditory, motor control, and cognition (LeRouge et al. 2013), which technology providers should consider when designing a product or service for the elderly group. The changes are very different for each individual, so it is not possible to point to a specific level and generalize that for all elderly. Some elderly (i.e. early retirees) are really agile and have great motor control, while their auditory sense does not function very well. Others may have great cognition but motor control is not completely in function (Nelson and Dannefer 1992). Thus, there is a need for special design of technology tools for this diverse group (Clarkson et al. 2013).

Assistive technology can, to some extent, compensate for the declined functionality of elderlies’ physical and cognition (Pressler and Ferraro 2010). Tele-health technology can monitor elderly at home and reduce the number of doctor visits (Lin et al. 2013), which makes life more convenient for elderly individuals and would save costs for healthcare providers. Communication technologies such as tablets, smart phones, and computers have facilitated social interactions from distance (Gao et al. 2015). This way, family members and friends have been able to keep in touch more often without travelling back and forth for each visit. The wireless sensors and distance monitoring assist healthcare providers to monitor the physical and psychological situation of the elderly and speed up their service and help (Jeong et al. 2010). There are on-going projects in Sweden to help with such technology, such as ACTION, which stands for Assisting Carers using Telematics Interventions to meet Older people’s Needs (Magnusson and Hanson 2012). This project is running in 25 municipalities in Sweden using technology to provide many services to elderly (Magnusson and Hanson 2012).

In Sweden, the majority of people have access to a computer and the Internet, and the Internet Foundation in Sweden has published that 69 % of the whole population uses the Internet on a daily basis (Findahl 2011). In 2011, statistics showed that 64 % of elderlies aged between 55 and 64 visits the Internet every day, while 51 % of early retirees (aged between 65 and 74) and only 22 % of those aged more than 75 visit the Internet on a daily basis. These numbers confirm the use of technology by the elderly and the diversity among them. There is an incline of visits to social networks among the elderly in Sweden. About half of the Internet users aged between 55 and 64 is member of social networks. Among young retirees (65–74) only 36 % visit social networks. The oldest group (75+) had the highest increase from 12 % in 2010 to 31 % in 2011 for involvement in social networks (Findahl 2011).

Despite the availability and access to technology, extant literature has identified several barriers for elderly using technology tools, namely attitudinal (Ellis and Allaire 1999), cognitive (memory and speed of processing) (Czaja et al. 2006), privacy concern (Caine et al. 2006), and security and safety (Miskelly 2001). The study of Werner et al. (2011) shows that not only physical age-related changes but also psychological wellbeing and life course events, such as loss of spouse and retirement, have a significant impact on the use of technology tools.

The literature illustrates several theories for explaining and predicting the technology acceptance behaviour, such as theory of reasoned action (TRA), technology acceptance model (TAM), and the unified theory of acceptance and use of technology (UTAUT).

TRA was developed by Fishbein and Ajzen (1975) and focused on intention as the main determinant of a person’s actual behaviour. Attitude toward a behaviour and subjective norm or social pressure were introduced as two constructs influencing intention. TRA explains human behaviour in general and it is not limited to a specific context (Davis et al. 1989).

Davis et al. (1989) developed and introduced TAM and it adapted TRA with the specific context of computer-based information systems (Davis et al. 1989). Two main constructs of Perceived Usefulness (PU) and Perceived Ease of Use (PEOU) replaced other measures in TRA.

In continuous of improvement of user acceptance models, Venkatesh et al. (2003) developed UTAUT after reviewing eight different models. This model included more variables such as performance expectancy and social influence, plus four moderators, namely age, gender, experience, and voluntariness of use, which makes it a stronger model for predicting user behaviour.

TAM and UTAUT has been widely applied to different contexts and have become a robust and powerful model for explaining user acceptance in different technological contexts (Venkatesh and Davis 2000; Wu and Lu 2013; Harms et al. 2014). However, they do not include the age-related attributes.

Chen and Chan (2014) tested the gerontechnology usage behaviour in TAM using attitudinal factors with a sample of 1012 seniors aged 55 and above in Hong Kong. China has the largest percentage of older population in the world and Hong Kong has the second longest life expectancy at birth with 87.2 years (Chen and Chan 2014). They called their model Senior TAM (STAM). In Spain, TAM has been used for the elderly and use of the Internet with two additional constructs: perceived enjoyment from TAM3 (Venkatesh and Bala 2008) and result demonstrability from TAM2 (Ramón-Jerónimo et al. 2013). Mitzner et al. (2010) conducted 18 focus groups with 113 elderlies and tested the older adults talk technology usage and attitudes in the US. The result of their study demonstrated a positive attitude of elderlies towards technology and suggested that focusing on the benefits of technology tools in education and training programs may improve anticipated technology adoption. Another study in the US investigates the technology usage in general and frequency of use among elderly (Olson et al. 2011). Their study shows that the differences among younger adults and older adults in using technology depend on the technology domain; however, in general, younger adults use a larger variety of technologies in comparison with older adults.

**Fig. 1:**
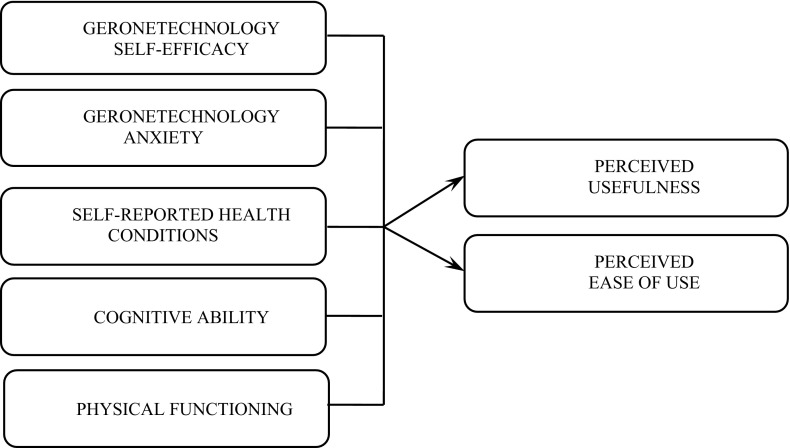
Conceptual Framework

Based on the previous studies, we proposed a research model that includes age-related factors from STAM as antecedent conditions and PU and PEOU as outcomes. Figure 1 demonstrates the research proposal.

The proposed research model in this study includes the following gerontechnology characteristics (also known as age-related attributes): cognitive abilities (CA), gerontechnology self-efficacy (SE), gerontechnology anxiety (ANX), self-reported health condition (SF), and physical functioning (IADL). Cognitive ability (CA) refers to the degree to which an elderly has the ability to think, learn, memorize, and concentrate (adopted from Chen and Chan 2014). Gerontechnology self-efficacy (SE) refers to the degree to which an elderly feels he/she is able to use technology tools that can increase independent living and social participation of the elderly in relatively good health, comfort, and safety (adapted from UTAUT). Gerontechnology anxiety (ANX) explains the degree to which an elderly’s nervousness when he/she is faced with the possibility of using a technology tool that is supposed to improve the independence and involvement in social activities of the elderly (adapted from UTAUT). Self-reported health conditions (SH) measured the degree to which the elderly considers their general health status and a comparison with their same age group (adapted from Chen and Chan 2014). Physical functioning (IADL) describes the degree to which the elderly can independently perform instrumental activities in their everyday life (adapted from Chen and Chan 2014).

The model tests the effect of each one of the abovementioned age-related variables on two outcomes, which are perceived ease of use and perceived usefulness. Perceived ease of use (PEOU) refers to the degree to which the use of a technology tool becomes useful and the soonest that an elderly believes the use is effortless (adapted from Venkatesh and Davis, 2000). Perceived usefulness (PU) is considered the degree to which an elderly believes that employing a specific technology tool would improve the performance of his/her life quality (adapted from Davis et al. 1989).

The existing literature shows that STAM has not been widely adopted, examined, replicated, and validated at all. Thus, the following propositions are formulated:

### **Proposition 1**

*Gerontechnology characteristics have a positive significant impact on senior citizens’ perceived usefulness.*

### **Proposition 2**

*Gerontechnology characteristics have a positive significant impact on senior citizens’ perceived ease of use.*

## Method

### Size and nature of the sample

Based on the literature, we developed a questionnaire in the English language. After minor revisions, based on comments from five researchers, an expert in the field and language translated the questionnaire into the Swedish language. To ensure the quality of translation, an expert back translated the Swedish version into English. The result was satisfactory as it matched the original English version. Thereafter, we ran a pilot test with four researchers and five elderlies. A link to the final version of the questionnaire was e-mailed to 1000 elderlies, with 610 completing the questionnaire, resulting in a response rate of 61 %. In parallel, face-to-face data gathering from the elderly in town was conducted. Among 400 elderly who were approached, 201 completed answers were received, representing a 50 % response rate. In this way, the sample has a variety of technology users and it is not gathered only from those who already employ the Internet and e-mail in their everyday life. In total, only 2 % of respondents had never used e-mail, showing that even the majority of respondents from the offline survey used e-mail.

Statistics show that (Findahl 2011) the majority of the population and even elderly use the Internet on a daily basis; therefore, we believe that the online survey would change the results tremendously in comparison with the offline data gathering method.

The total of 811 elderly answered the questionnaire online and offline. Among them, 50.4 % were female and 83 % were 65–74 years old. There had been a debate about the retirement age in Sweden; however, the more individuals work the higher their retirement salaries will become. The Aging Report by European Economy shows a retirement age of 61–67 years old for Sweden (European Economy 2009).

The descriptive statistics show that about 70 % lived with a household member, approximately 36 % had university and higher education, about 72 % were married or had a partner, about 70 % were retired, and about 49 % had a yearly salary of less than 250,000 SEK. Table 1 illustrates the characteristics of respondents.

**Table 1:** Respondents’ characteristics

Characteristics	Percentage
Age	
60–64	8.4
65–74	84.0
75–84	2.8
85 or more	0.4
Gender	
Female	50.4
Male	49.6
Marital status	
Married	58.3
Partner	13.3
Never married	5.1
Divorced/separated	13.8
Widowed	9.5
Living arrangement	
With a household member(s)	69.5
Living alone at home	30.2
In a nursing house	0.2
Work status	
Full-time work	18.2
Part-time work	8.1
Retired	71.4
Not applicable/never worked	2.2
Primary means of living	
Salary/wages	23.4
Retirement wages/benefits	73.6
Property income	1.2
Local government or community subsidy	1.6
Spouse/child/grandchild/relative(s) support	0.1
Economic status (annual thousand SEK)	
Less than 150	11.1
151–200	19.5
201–250	18.5
251–300	14.9
301–350	12.2
More than 351	23.8

The questionnaire was designed in several sections. First, general use of technology that focuses on technologic tools that support elderlies’ independence, improve their quality of life and bring safety and comfort to their everyday life. These technology tools were divided into four categories: housing, communication, health technology, and education. In the very first section of the questionnaire respondents indicated about the technology tolls in each category, if they “have never heard of” or “have heard but not used” or “have been using or used.” Responses illustrated that the elderly had heard or used almost all of the technology tools except “tele-care.” Among respondents, 41 % had never heard about distance healthcare. This confirms the high availability of different technology tools in Sweden.

The second section of the questionnaire focused on the acceptance of the technology. Questions about health and ability characteristics were in third section. Demographic characteristics were in the final part of the questionnaire. Before the final section, there were originally four additional sections, which we did not use in this specific study as it is part of a larger project. The questionnaire ended with some space for comments and suggestions. The majority of respondents explained that it was interesting to answer the questions.

### Measurement and scales

The questionnaire was designed in English and passed several content validity tests. It was translated into the Swedish language and after another round of content validity the questionnaire was translated back to English, which ended up being similar to the original English questionnaire. The final version in the Swedish language went through content validity with three researchers and five elderlies. Respondents received an e-mail with a link to the online questionnaire.

Table 2 illustrates all the constructs and items used to measure them. All items were adapted from previous research and the references are available in Table 2. All items were measured on a 10-point Likert Scale (1 = disagree strongly to 10 = agree strongly) along with a visual analogue format that was a graphic scaling. Wee et al. (2008) suggest that elderlies will have an optimal range of response categories on a 10-point scale. Additionally, this is the preferred scale for higher validity and reliability.

**Table 2:** List of variables, items, and sources of measurements

Variable name	Items	Adopted from
Perceived Usefulness (PU)	PU1—Using technology enhances my effectiveness for daily activities	Davis et al. (1989)
	PU2—Using technology makes my life more convenient	
	PU3—I find technology useful for daily activities	
	PU4—Using technology improves my performance in daily activities	
	PU5—Using technology makes it easier to do my daily activities	
Perceived ease of use (EOU)	PEOU1—I find technology easy to use	Davis et al. (1989)
	PEOU2—I am skilful at using technology	
	PEOU3—I find technology flexible for daily activities	
	PEOU4—My interaction with technology is clear and understandable (without confusion)	
Gerontechnology self-efficacy	SE1—I could complete a task using technology if there was someone to demonstrate how	Venkatesh et al. (2003)
	SE2—I could complete a task using technology with just the instruction manual for assistance	
Gerontechnology anxiety	ANX1—I feel apprehensive about using the technology	Venkatesh et al. (2003)
	ANX2—I hesitate to use the technology for fear of making mistakes I cannot correct	
Cognitive ability	CA1—How satisfied are you with your ability to learn new information?	Chen and Chan (2014)
	CA2—How well are you able to concentrate?	
	CA3—How satisfied are you with your ability to make decisions?	
Physical function	IADL1—bility to use telephone	Chen and Chan (2014)
	IADL2—Grocery shopping	
	IADL3—Food preparation	
	IADL4—Doing housework or handyman work	
	IADL5—Laundry	
	IADL6—Getting to places beyond walking distance	
	IADL7—Taking medications	
	IADL8—Managing money	
Self-reported health conditions	SH1—How are your general health conditions?	Chen and Chan (2014)
	SH2—How is your health condition compared with the same age groups?	
	SH3—How good is your hearing?	
	SH4—How well can you see?	
	SH5—How well are you able to move around?	

#### Outcomes

The two outcomes in this study are perceived ease of use (PEOU) and perceived usefulness (PU). These two outcomes are widely used in TAM (Davis et al. 1989) and STAM has included them as well (Chen and Chan 2014). As Table 2 shows, PU is measured with 5 items and PEOU with 4 items.

#### Antecedent conditions

Antecedent conditions in this study are: cognitive abilities (CA) measured with 3 items, gerontechnology self-efficacy (SE) measured with 2 items, gerontechnology anxiety (ANX) measured with 2 items, self-reported health condition (SH) measured with 5 items, and physical functioning (IADL) measured with 8 items.

### Proposition testing and results

To test propositions, we follow a set-theoretic approach using fsQCA. fsQCA is an analytical tool that examines how alternative conditions of causes (i.e., antecedents or drivers) combine together and contribute to high membership scores of the outcome of interest (Rihoux and Ragin 2009). fsQCA models the concept of conjunctural causation; rather than a single net effect, it provides possible combinations of various causal conditions that can be linked to the same outcome (Schneider et al. 2010). Thus, it captures the concept of equifinality (Fiss 2011). fsQCA solutions provide the necessary (i.e., conditions that produce the outcome but by themselves may not be enough) and sufficient (i.e., conditions that always lead to the outcome) conditions that are associated with the outcome (Ragin 2000, 2008).

The present study aims to examine how conditions of the STAM model (e.g., SE: gerontechnology self-efficacy; ANX: gerontechnology anxiety; SH: self-reported health conditions; CA: cognitive ability; and IADL: physical functioning) affect senior Swedish consumers’ perceived usefulness (PU) and perceived ease of use (PEOU). To test propositions, we proceeded in three steps. First, we calibrated all involved variables into fuzzy sets. Fuzzy sets take values between 0 and 1 and signify the degrees of membership in a specific category (Skarmeas et al. 2014). Values of 0, 0.50, and 1 signify non-membership, the point of maximum ambiguity, and full membership, respectively (Woodside and Zhang 2013). Drawing on Ragin (2000, 2008) we use three breakpoints for set calibration: 0.05 (i.e., full non-membership), 0.50 (i.e., crossover point), and 0.95 (i.e., full membership).

The second step of the fsQCA analysis involves the development of the truth table, with *2* ^*k*^ rows; *k* represents the number of causal conditions used in the analysis (Crilly 2011). The present study involves *2* ^*5*^ possible combinations for PU and an equal number of possible combinations for PEOU. Each row on the truth table shows combinations of causes and the entire table represents all the possible combinations of conditions for the outcome of interest (Fiss 2011). To identify possible relevant combinations that are associated with at least one observation between the predictors and the outcome, the truth table needs to be reduced. Two criteria are used for reducing the truth table: (1) minimum number of cases needed so a solution can be extracted; and (2) minimum consistency levels needed for a particular solution to be meaningful (Ragin 2000). Consistency refers to the “degree to which a combination of causal conditions is reliably associated with the outcome” (Crilly 2011, p. 705). The minimum recommended threshold of consistency is 0.75 (Ragin 2008).

The third step comprises the simplification of the derived combinations of causes with the outcome of interest. fsQCA uses Boolean algebra and algorithms (i.e., truth table algorithm) to do so (Ragin 2000). To assess the derived solutions, fsQCA provides three possible outcomes: complex, intermediate, and parsimonious (Rihoux and Ragin 2009). The present study adopts complex solutions. Complex solutions make no particular simplifying assumptions, include neither easy nor difficult counterfactuals, and are suitable for a smaller number of causal conditions (Skarmeas et al. 2014; Elliott 2013). Coverage and consistency statistics are used to interpret fsQCA findings (Ragin 2008). Coverage captures the empirical importance of derived solutions in reaching the outcome of interest, illustrating how much of the outcome is captured by each solution pathway and by the entire solution (Fiss 2011). A model solution is explanatory when coverage ranges between 0.25 and 0.65 (Ragin 2008).

Table 3 shows the derived complex solutions for high degrees of PU and PEOU. The fsQCA findings derived two pathways of complex solutions for both PU and PEOU. All solutions exhibit acceptable consistency (i.e., ≥.80) and explain a satisfactory amount of cases in high financial performance (i.e., ≥.25 coverage ≤.65). For high memberships of PU, the first pathway indicates a combination of high levels of SE and SH, and low degrees of ANX and IADL. This pathway is consistent (consistency = 0.89) and explains a satisfactory amount of cases in PU (coverage = 0.28). The second solution recipe identifies a combination of high degrees of SE and CA and low levels of ANX and SH. This pathway is also consistent (consistency = 0.91) and explains an adequate amount of cases of high PU (coverage = 0.46). It can be concluded that high levels of SE and low levels of ANX appear in both solution pathways; thus, SE and ANX are considered necessary conditions—though not sufficient on their own—to obtain high PU. While SE and ANX conditions are “core” for high PU, SH, CA, and IADL play a peripheral role for reaching the outcome.

**Table 3:** Conditions for perceived usefulness and ease of use

Complex solution	Raw coverage	Unique coverage	Consistency
Perceived usefulness (PU)			
Model: f_pu = f(f_se, f_anx, f_sh, f_ca, f_iadl)			
se_cal* ~ anx_cal*sh_cal* ~ iadl_cal	0.276516	0.077922	0.891186
se_cal* ~ anx_cal* ~ sh_cal*ca_cal	0.316080	0.117486	0.903316
Solution coverage: 0.394003; solution consistency: 0.878864			
Frequency cutoff: 3.000000; consistency cutoff: 0.908194			
Perceived ease of Use (PEOU)			
Model: f_peou = f(f_se, f_anx, f_sh, f_ca, f_iadl)			
~ anx_cal* ~ sh_cal*ca_cal	0.387737	0.135728	0.907356
se_cal* ~ anx_cal*ca_cal* ~ iadl_cal	0.325287	0.073277	0.919727
Solution coverage: 0.461014; Solution consistency: 0.904751			
Frequency cutoff: 3.000000; Consistency cutoff: 0. 0.919282			

*se* geronetechnology self-efficacy, *anx* geronetechnology anxiety, *sh* self-reported health conditions; ca cognitive ability, *iadl* physical functioning

Similarly, for high memberships of PEOU the first solution recipe demonstrates a combination of high levels of CA, and low degrees of ANX and SH. The first pathway is fairly consistent (consistency = 0.91) and explains a satisfactory amount of cases in PU (coverage = 0.39). The second pathway solution shows a combination of high degrees of SE and CA, and low levels of ANX and IADL. This recipe is also consistent (consistency = 0.92) and explains an adequate amount of cases with degrees of PEOU (coverage = 0.32). It is evident that high levels of CA and low levels of ANX appear in both solution pathways and are necessary conditions—though not sufficient on their own—for high memberships of PEOU. On the other hand, SE, SH, and IADL are peripheral for reaching high PEOU. Drawing on Ragin and Fiss (2008), Table 4 illustrates the notation system for fsQCA solutions for high memberships of PU and PEOU. Full black circles (**•**) indicate the presence of a condition; large and small circles indicate core and peripheral conditions, respectively.

**Table 4:** Ideal configurations for perceived usefulness and ease of use

	Perceived usefulness (PU)	Perceived ease of use (PEOU)
Geronetechnology self-efficacy	●	•
Geronetechnology anxiety	**●**	**●**
Self-reported health conditions	**•**	**•**
Cognitive ability	**•**	**●**
Physical functioning	**•**	**•**

Black circles indicate the presence of a condition; large circles indicate core conditions; small circles indicate peripheral conditions

## Conclusion

This study examined the elderly acceptance of technology tools in Sweden, by incorporating age-related attributes into TAM and testing STAM. This way, the biophysical and psychological abilities and issues that the elderly experience from using technology tools in everyday life have been considered thoroughly.

Results show that all the age-related attributes (i.e., SE, ANX, SH, CA, and IADL) are important to perceiving that a technology tool is easy to use and is useful; however, some play a core role in this relation. ANX and CA are core essentials for achieving high PEOU, while SE and ANX are core for PU. The study of Chen and Chan (2014) has also shown the strong predictive power of SE.

The conclusion is that the elderly’s apprehension for facing technology tools is a major concern considering both ease of use and usefulness of a technology. The healthcare providers, technology designers, and family members should understand these feelings and anxieties. Professional psychologists might be able to provide guidelines for dealing with these emotions.

The other major factor for PU is SE, meaning that elderlies’ understanding and feelings about their knowledge and skills determine how they consider the usefulness of a technology tool. The findings of Chen and Chan (2014) also show that confidence in using technology tools leads to higher levels of PU and PEOU. In order to improve the levels of knowledge, technology providers might have some solutions. However, it terms of feelings, a psychologist might have a specific guideline for the elderly.

Finally, CA was identified as a core actor for higher levels of PEOU. This refers to the elderlies’ ability to concentrate, memorize, learn, and think as a main indicator for considering ease of use of a technology tool. This relates to a very special characteristic of the elderly that varies a lot within their large and diverse group. The sooner and more often individuals use their cognitive abilities, the more powerful and agile it would become. On the other hand, those who are over 85 years old are the ones who need more help and attention.

This study suggests both theoretical and managerial implications. First, by adopting STAM this research adds to the body of literature, since it has been tested in Sweden for the very first time. Second, the analysis method is very unique and fsQCA has been applied to STAM for the very first time. Third, findings about the core variables for PU and PEOU shed light on major constructs in the literature about the elderly.

The findings reveal clear implications for decision makers in the government body, technology designers, healthcare providers, and elderlies’ family members. All the age-related factors play a role in acceptance of technology; thus, each one of them (i.e., cognitive abilities, gerontechnology self-efficacy, gerontechnology anxiety, self-reported health condition, physical functioning) should be taken into consideration when providing healthcare services and designing technology tools for this group. The following implications are suggested for practitioners. First, understand, respect, and deal with the anxiety and emotions toward elderlies’ use of technology tools. Second, practitioners need to consider the elderlies’ self-understanding of their knowledge and skills for using technology tools. They might need to find ways to improve this self-image among the elderly in order to motivate the use. Finally, technology designers have to completely incorporate the special needs of the elderly in their designs. Healthcare providers and family members need to work on the elderlies’ cognitive abilities.

The case of Sweden has very specific characteristics, as explained in the introduction; the majority of people are highly educated and have access to technology tools. These features build a platform that speeds up the technology acceptance. On the other hand, the results of this study show that self-understanding about skills and knowledge plus anxiety about using technology are still two major factors for elderly.

Despite the important insights found in this study, we acknowledge that it has limitations. We employed an online survey that excludes non-internet users from the sample. Thus, we drew our analysis from a specific group of the elderly who already use the Internet. Future studies may use offline means of data gathering to see if there are any differences in the relationships between antecedent conditions and outcomes. Furthermore, we did not incorporate attitude and intention to use in the research model, since the focus was on gerontechnology and PU and PEOU. However, including attitude and intention to use would add insight and understanding about the use of technology tools by the elderly. Finally, the context in this study was Sweden, which has an infrastructure for technology tools that are widely available for Sweden’s inhabitants. Testing the research model in other countries would add to the body of literature.
